# First-Line Durvalumab–Tremelimumab in Advanced Hepatocellular Carcinoma: Real-World Data from Turkey

**DOI:** 10.3390/jcm15155997

**Published:** 2026-08-01

**Authors:** Mustafa Murat Mıdık, Gökhan Şahin, Bekir Mert Durukan, Fatih Kuş, Kübra Haşimoğlu Gürün, Sinan Ünal, Cem Mirili, Canan Karan, Engin Hendem, Teoman Şakalar, Miray Aydoğan, Bahadır Köylü, Nadiye Sever, Bahattin Engin Kaya, Tülay Kuş, Canberk Şencan, Atike Pınar Erdoğan, Hatime Arzu Yaşar, Hilal Karakaş, Oğuzhan Yıldız, Bahiddin Yılmaz, Murat Alan, Bülent Çetin, Nedim Turan, Melek Karakurt Eryılmaz, Mehmet Artaç, İlkay Tuğba Ünek, Fatih Selçukbiricik, Mehmet Ali Şendur, Hasan Çağrı Yıldırım, Şuayib Yalçın

**Affiliations:** 1 Department of Medical Oncology, Ege University Medical Faculty, 35100 Izmir, Turkey; gokhan.sahin@ege.edu.tr (G.Ş.); hasan.cagri.yildirim@ege.edu.tr (H.Ç.Y.); 2 Department of Medical Oncology, Hacettepe University, 06800 Ankara, Turkey; mertdurukan@hacettepe.edu.tr (B.M.D.); syalcin@hacettepe.edu.tr (Ş.Y.); 3 Department of Medical Oncology, Ankara Sanatorium Hospital, 06290 Ankara, Turkey; fatihkush@hotmail.com; 4 Department of Medical Oncology, Ondokuz Mayıs University, 55270 Samsun, Turkey; drkubrahasimoglu@gmail.com (K.H.G.); bahiddin.yilmaz@omu.edu.tr (B.Y.); murat.alan@samsun.edu.tr (M.A.); bulent.cetin@omu.edu.tr (B.Ç.); 5 Department of Medical Oncology, Faculty of Medicine, Atatürk Training and Research Hospital, Izmir Katip Çelebi University, 35620 Izmir, Turkey; drsinanunal@gmail.com; 6 Department of Medical Oncology, Adana Middle East Hospital, 01140 Adana, Turkey; cemirili@gmail.com; 7 Department of Medical Oncology, Denizli Tekden Hospital, 20010 Denizli, Turkey; karancanan16@gmail.com; 8 Department of Medical Oncology, Mengücek Gazi Training and Research Hospital, Binali Yıldırım University, 24002 Erzincan, Turkey; enginhende61@gmail.com; 9 Department of Medical Oncology, Kahramanmaraş Necip Fazıl City Hospital, 46050 Kahramanmaraş, Turkey; drteomansakalar@gmail.com; 10 Department of Medical Oncology, Kartal Dr. Lütfi Kırdar City Hospital, 34865 Istanbul, Turkey; mirayaydogan1991@gmail.com (M.A.); turan.nedim@hotmail.com (N.T.); 11 Department of Medical Oncology, Faculty of Medicine, Koç University, 34450 Istanbul, Turkey; bkoylu@ku.edu.tr (B.K.); fselcukbiricik@ku.edu.tr (F.S.); 12 Department of Medical Oncology, Haydarpaşa Sample Training and Research Hospital, 34668 Istanbul, Turkey; dr.nadya@hotmail.com; 13 Department of Medical Oncology, Meram Faculty of Medicine, Necmettin Erbakan University, 42090 Konya, Turkey; bekaya@erbakan.edu.tr (B.E.K.); dr.oguzhan@outlook.com (O.Y.); mkeryilmaz@erbakan.edu.tr (M.K.E.); maytac@erbakan.edu.tr (M.A.); 14 Department of Medical Oncology, Faculty of Medicine, Gaziantep University, 27410 Gaziantep, Turkey; drtulaykus83@hotmail.com; 15 Department of Medical Oncology, Faculty of Medicine, Dokuz Eylül University, 35220 Izmir, Turkey; sencan.canberk@deu.edu.tr (C.Ş.); tugba.gun@deu.edu.tr (İ.T.Ü.); 16 Department of Medical Oncology, Hafsa Sultan Hospital Medical Oncology, Celal Bayar University, 45140 Manisa, Turkey; atike.erdogan@cbu.edu.tr; 17 Department of Medical Oncology, Faculty of Medicine, Ankara University, 06070 Ankara, Turkey; hayasar@ankara.edu.tr; 18 Department of Medical Oncology, Ankara Bilkent City Hospital, 06800 Ankara, Turkey; hilal.karakas@ankara.edu.tr (H.K.); masendur@yahoo.com.tr (M.A.Ş.)

**Keywords:** tremelimumab, durvalumab, hepatocellular carcinoma, immunotherapy, real world, safety

## Abstract

**Background:** Hepatocellular carcinoma (HCC) is one of the leading causes of cancer-related mortality worldwide. Although the STRIDE regimen (durvalumab plus tremelimumab) has demonstrated an overall survival benefit in clinical trials, real-world evidence remains limited, particularly in Turkish clinical practice. This study aimed to evaluate the effectiveness and safety of first-line STRIDE therapy in patients with unresectable HCC treated in routine clinical practice. **Methods:** We conducted a retrospective multicenter study including patients with unresectable HCC who received first-line durvalumab plus tremelimumab through the Turkish national Early Access Program across 18 oncology centers. Progression-free survival (PFS) and overall survival (OS) were estimated using the Kaplan–Meier method, and exploratory Cox regression analyses were performed to evaluate baseline prognostic factors. **Results:** Thirty-two patients were included. The median PFS was 7.25 months (95% CI, 3.68–22.29), and the median OS was 11.43 months (95% CI, 4.50–not reached). The underlying liver disease etiology was non-viral in 53.1% of patients, hepatitis B virus in 40.6%, and hepatitis C virus in 6.3%. Grade ≥ 3 treatment-related adverse events occurred in fewer than 10% of patients. **Conclusions:** In this multicenter real-world cohort, first-line durvalumab plus tremelimumab appeared to be a feasible treatment option and was generally well tolerated in patients with unresectable HCC. However, given the retrospective design, small sample size, and absence of a control group, these findings should be interpreted cautiously and require confirmation in larger prospective comparative studies.

## 1. Introduction

Hepatocellular carcinoma (HCC) is the most common primary liver malignancy and one of the leading causes of cancer-related mortality worldwide. Despite advances in surveillance and treatment, most patients are diagnosed with unresectable or advanced-stage disease, resulting in poor long-term survival. The Barcelona Clinic Liver Cancer (BCLC) staging system remains the standard framework for treatment allocation, with systemic therapy recommended for patients with advanced disease and for selected patients with intermediate-stage disease who are unsuitable for further locoregional treatment or have become refractory to transarterial chemoembolization (TACE) [1,2,3,4,5].

The treatment landscape for unresectable HCC has evolved rapidly with the introduction of immune checkpoint inhibitor (ICI)-based combinations. For patients who are eligible for immunotherapy, atezolizumab plus bevacizumab, nivolumab plus ipilimumab, and durvalumab plus tremelimumab (STRIDE regimen) are currently recommended first-line treatment options according to international guidelines [5,6,7,8]. Among these, the HIMALAYA trial demonstrated a significant overall survival benefit for the STRIDE regimen compared with sorafenib while maintaining a favorable safety profile, establishing STRIDE as an important first-line therapeutic option [9,10].

Although randomized clinical trials provide high-quality evidence, their highly selected patient populations may not fully reflect routine oncology practice. Real-world data are therefore essential to evaluate treatment effectiveness and safety in broader and more heterogeneous patient populations encountered in daily clinical care. This is particularly relevant in Turkey, where access to the STRIDE regimen has been largely restricted to a national early access program because reimbursement has not yet been widely available. Consequently, patient selection and treatment patterns may differ from those observed in pivotal clinical trials.

Furthermore, real-world evidence regarding first-line STRIDE therapy remains limited, particularly in Middle Eastern and Mediterranean populations. Multicenter data describing the clinical outcomes of Turkish patients treated with durvalumab plus tremelimumab are currently lacking.

Therefore, we conducted this multicenter retrospective study to describe the effectiveness and safety of first-line durvalumab plus tremelimumab in Turkish patients with unresectable hepatocellular carcinoma treated in routine clinical practice.

## 2. Materials and Methods

### 2.1. Study Design and Patient Population

This retrospective multicenter study was conducted across 18 oncology centers in Turkey. Patient records were screened between January 2020 and December 2024. Eligible patients received first-line durvalumab plus tremelimumab only after the regimen became available through the Turkish national Early Access Program (EAP). Therefore, although the study database covered the period from 2020 to 2024, initiation of STRIDE treatment occurred only after implementation of the EAP. Consecutive eligible patients treated during the EAP period were included in the analysis. Eligible patients were required to meet all of the following inclusion criteria: (1) age ≥ 18 years; (2) radiologically and/or histologically confirmed unresectable hepatocellular carcinoma; (3) receipt of first-line durvalumab plus tremelimumab through the Turkish national Early Access Program; (4) Child–Pugh class A or selected class B liver function, according to the treating physician’s judgment; and (5) availability of complete baseline clinical characteristics and follow-up data. Patients were excluded if they (1) had received previous systemic therapy for unresectable HCC; (2) had insufficient clinical data for efficacy or safety assessment; or (3) were followed for less than one month after treatment initiation. Patients with a follow-up duration of less than one month were excluded because adequate clinical and radiological data were unavailable for reliable assessment of treatment response, progression-free survival, and treatment-related adverse events.

All patients received durvalumab plus tremelimumab through the Turkish national Early Access Program. As required by the Early Access Program and institutional regulations, written informed consent for treatment and the scientific use of anonymized clinical data had been obtained before treatment initiation.

Before initiation of systemic therapy, all patients underwent upper gastrointestinal endoscopy as part of routine clinical practice to evaluate the presence of gastroesophageal varices in accordance with standard management of patients with cirrhosis and unresectable hepatocellular carcinoma. This evaluation was performed as part of routine patient care and was not a study-specific procedure.

The STRIDE regimen is termed as 300 mg of tremelimumab for one dose plus 1500 mg of durvalumab every 4 weeks until progression or intolerable adverse events.

Locoregional therapies, such as transarterial chemoembolization (TACE) and transarterial radioembolization (TARE), were administered prior to the STRIDE regimen. Patients with BCLC stage B were eligible only if they were considered unsuitable for further locoregional therapy after multidisciplinary evaluation. Indications for first-line systemic treatment included TACE-refractory disease, TACE-unsuitable disease due to extensive intrahepatic tumor burden, diffuse or bilobar liver involvement, impaired liver function precluding further locoregional therapy, or lack of expected clinical benefit from additional locoregional treatment. Consequently, all enrolled patients represented an unresectable HCC population receiving first-line systemic therapy according to contemporary clinical practice.

Radiological assessments were performed approximately every 8–12 weeks.

The study protocol was approved by the institutional ethics committee on October 30, 2025 (approval number: 25-10.2T/56), and this study was conducted in accordance with the Declaration of Helsinki.

### 2.2. Statistical Analysis

Baseline features extracted from the shared database included demographic characteristics, liver disease etiology, cirrhosis status, ECOG-PS, alpha-foetoprotein (dichotomized at 400 ng/mL), and tumor burden features (size of HCC lesions, macrovascular invasion, extrahepatic spread, and Barcelona Clinic Liver Cancer [BCLC] stage). Treatment-related variables included the dates of drug administration, treatment interruptions, or discontinuation.

Categorical variables are reported as counts and percentages, and continuous variables are reported as medians with ranges. Associations between categorical variables were assessed with the Chi-square test, whereas comparisons of continuous variables were performed using the Student’s *t* test or Mann–Whitney U test, as appropriate. The PFS was defined as the interval from tremelimumab and durvalumab initiation to either disease progression or death, and overall survival (OS) as the time from treatment start to death from any cause. Tumor response was evaluated according to RECIST v1.1 criteria. The objective response rate (ORR) was defined as the proportion of patients achieving complete response (CR) or partial response (PR), while the disease control rate (DCR) included CR, PR, or stable disease (SD). Survival probabilities were estimated by the Kaplan–Meier method. Prognostic factors influencing OS were examined through univariable and multivariable Cox proportional hazards models; variables with *p* < 0.20 in univariable testing or considered clinically relevant were included in multivariable analysis. Hazard ratios (HRs) and 95% confidence intervals (CIs) are reported. All analyses were performed using SPSS Statistics version 26 (IBM Corp., Armonk, NY, USA), and statistical significance was set at a two-sided *p* < 0.05.

Adverse events (AEs) were graded according to CTCAE v5.0. Time-to-toxicity was defined as the interval between treatment initiation and the first occurrence of each AE. Temporal toxicity patterns were analyzed using kernel density estimation to derive smoothed hazard functions expressed as events per patient-month. Hazard functions were visualized as individual curves and heatmaps (0–15 months) separately for all-grade and grade ≥ 3 AEs, with normalized intensities to facilitate comparisons.

## 3. Results

### 3.1. Patient Characteristics

This study included 32 patients with a median age of 64 years (range, 23–85 years), and the majority were male (68.8%). Most patients had a good performance status, with 87.5% having an ECOG performance status of 0–1. Liver function was generally well preserved, with 30 patients (93.8%) classified as Child–Pugh class A, while one patient had Child–Pugh B7 and one had Child–Pugh B8 liver function.

At treatment initiation, 11 patients (34.4%) had unresectable BCLC stage B disease and were considered unsuitable for further locoregional therapy because of multidisciplinary team assessment, while the remaining 21 patients (65.6%) had advanced BCLC stage C disease requiring first-line systemic treatment. Non-viral etiologies represented the largest subgroup (53.1%), followed by hepatitis B virus infection (40.6%) and hepatitis C virus infection (6.3%). Cirrhosis was present in 71.9% of patients.

Advanced disease characteristics were common. Macrovascular invasion was observed in 25.0% of patients, while 50.0% had extrahepatic metastases. The lung (21.9%) and bone (18.8%) were the most frequent metastatic sites. Most patients (75.0%) had tumors larger than 5 cm, and 34.4% had an AFP level ≥ 400 ng/mL. Nearly half of the cohort (46.9%) had received previous locoregional therapy (Table 1).

### 3.2. Safety

Treatment demonstrated a favorable safety profile. Adverse events of any grade occurred in 34.4% of patients, whereas grade 3 or higher toxicities were uncommon, occurring in only 9.4%. Treatment discontinuation due to toxicity was rare (6.3%), indicating that the regimen was generally well tolerated (Table 2). 

Among treatment-related adverse events, pruritus or rash was the most frequently observed toxicity, affecting 21.9% of patients. Immune-related endocrine adverse events included hypothyroidism (9.4%) and adrenal insufficiency (3.1%). Gastrointestinal toxicity, including diarrhea or colitis, occurred in 9.4%, while pneumonitis and elevated AST/ALT levels were each reported in 3.1% of patients. Overall, adverse events were predominantly manageable, with serious toxicities occurring infrequently (Table 3).

### 3.3. Effectiveness

**Figure 1:**
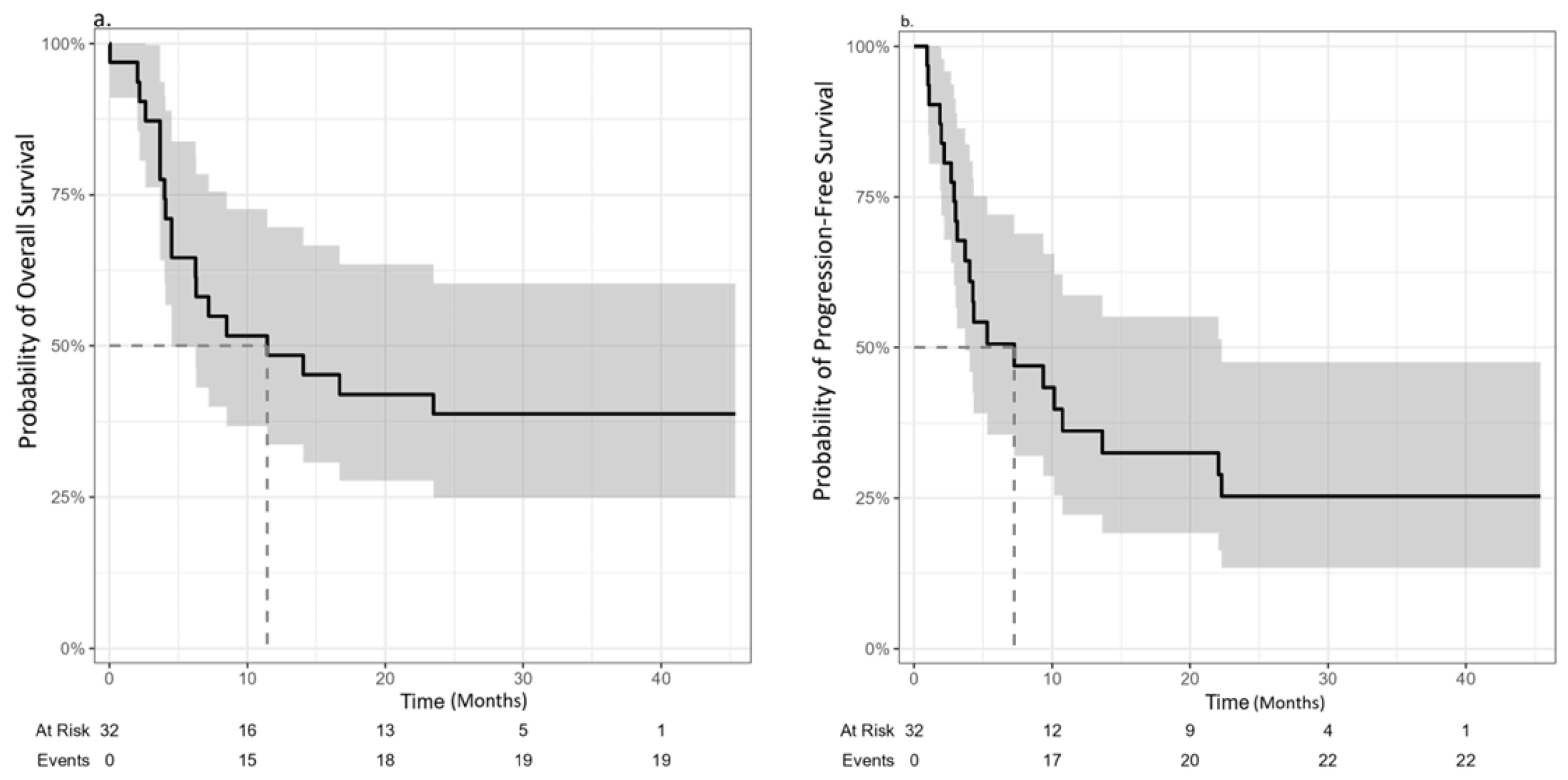
Kaplan–Meier curves for OS and PFS in the overall population of patients treated with durvalumab plus tremelimumab. (**a**) The median OS was 11.43 months (95% CI 4.5–NA); (**b**) the median PFS was 7.25 months (95% Cl 3.68–22.29). NA: Not applicable; OS: overall survival; PFS: progression-free survival; 95% Cls: 95% confidence intervals.

The median PFS was 7.25 months (%95 confidence interval (CI) 3.68–22.29), and the median OS was 11.43 months (%95 CI 4.5-NA) in the study population (Figure 1). 

Univariable Cox regression analysis did not identify any baseline clinical or disease-related factor that was significantly associated with progression-free survival (PFS) or overall survival (OS). Variables including sex, BCLC stage, disease etiology, macrovascular invasion, extrahepatic spread, lung or bone metastases, tumor size greater than 5 cm, AFP level ≥ 400 ng/mL, cirrhosis, prior locoregional therapy, and treatment-related adverse events were not significantly associated with survival outcomes (all *p* > 0.05). Although bone metastasis showed a trend toward poorer PFS (HR 2.55), this association did not reach statistical significance. No statistically significant associations were observed between the evaluated baseline clinical variables and progression-free survival or overall survival. However, these findings should be interpreted with caution because the relatively small sample size and limited number of events reduced the statistical power to detect potentially clinically meaningful prognostic associations (Table 4).

Among the 32 patients included in this study, the objective response rate (ORR) was 34.3%, with one patient (3.1%) achieving a complete response (CR) and 10 patients (31.3%) achieving a partial response (PR). Stable disease (SD) was observed in five patients (15.6%), resulting in an overall disease control rate (DCR) of 50.0%. In contrast, progressive disease (PD) was documented in 10 patients (31.3%) as the best overall response. At the time of data cutoff, 19 patients (59.4%) had died.

Overall, these findings indicate that first-line durvalumab plus tremelimumab achieved meaningful antitumor activity in this real-world cohort, with approximately one-third of patients experiencing an objective response and one-half achieving disease control. The observed response rates reflect the clinical outcomes achieved in this real-world cohort and should be interpreted descriptively, particularly given the retrospective study design and the absence of a comparator group (Table 5).

## 4. Discussion

When the treatment of metastatic/unresectable HCC in history was researched, the first systemic effective drug was sorafenib, which was an oral multikinase inhibitor of the vascular endothelial growth factor receptor, the platelet-derived growth factor receptor, and Raf [10,11]. Then, the IMbrave150 trial showed that the combination of atezolizumab plus bevacizumab was superior to sorafenib in terms of progression-free survival (PFS) and overall survival (OS) [12]. After the immune checkpoint inhibitor showed efficiency in HCC, the STRIDE and CheckMate 9DW trials were conducted. The STRIDE and Checkmate 9DW trials showed longer mOS (16.43 months vs. 23·7 months) and median durations of treatment (22.34 months vs. 30·4 months) than Imbrave150 (mOS: 19.2 months; median duration of treatment: 18.1 months). However, the STRIDE and Checkmate 9DW trials did not show statistical significance with respect to PFS. Safety data from trials showed that Imbrave150 had more grade 3–4 treatment-related serious adverse events than STRIDE and Checkmate9WD5 [13,14,15]. 

We reported the STRIDE regimen as a first-line treatment for uHCC in Turkey in this multicenter study. We evaluated the safety and efficacy of the STRIDE regimen in 32 patients. Our study showed longer median PFS (7.25 mo ve 3.78 mo) than the HIMALAYA trial [14]. Some real-world data of the STRIDE regimen from Japan and Italy reported that the mPFS was 3, 3.9, and 5.7 months [16,17]. Fujii et al. reported a mPFS of 6.8 months, which was closer to our study [18]. This probably resulted from the small population size and the locoregional therapies that were applied to %46.9 of patients.

ORR and DCR were reported to be similar to the HIMALAYA trials and relevant real-world data. The median OS was 11.43 months (%95 CI 4.5-NA), and it was shorter than in other studies. Prinzi et al. and Kournoutas et al. found that their mOS was 15.5 and 14.6 months [19,20]. Some real-world data reported that mOS was not reached [16,17,18]. Relatively preserved PFS, despite short OS, may suggest limited access to effective post-progression systemic therapies and a possible influence of underlying liver dysfunction in this real-world cohort.

Akarca et al. showed HBV infection, HCV infection, and non-viral composed %68.2 %17.2 and %14.6, respectively, of the etiology of hepatocellular carcinoma in the population of Turkey. This study also reported that the BCLC B-C stage accounted for half of the population. Therefore, our populations showed inconsistency with previous Turkish populations [21]. Our study’s populations do not represent HCC with HCV infection due to the low rate of infection. 

Our study showed similar demographic features, macrovascular invasion, and extrahepatic spread rates compared to the literature [16,17,18,19,21,22].

Sorafenib, lenvatinib, ramucirumab, cabozantinib, and regorafenib are suggested after progression following first-line immune checkpoint inhibitor therapy or tyrosine kinase inhibitor therapy [23,24,25,26,27,28,29]. In Turkey, sorafenib is almost the only second-line agent, because regorafenib and cabozantinib are not tolerable, and lenvatinib is not feasible. The shorter mOS may be due to the lack of second-line agents other than sorafenib.

As reported in other real-world data, we showed that prognostic factors, such as male gender, etiology of HCC, BCLC staging, and locoregional therapies, were not associated with statistically significant differences in PFS or OS in our study [16,17,18,19,21,22].

Several recent real-world studies have evaluated the effectiveness of first-line durvalumab plus tremelimumab in patients with unresectable HCC. Although the survival outcomes observed in our cohort are generally comparable with those reported by Fujii et al., Casadei-Gardini et al., and Kournoutas et al., direct comparisons should be interpreted cautiously because of important differences in study populations [16,18,19]. Variations in the proportion of patients with BCLC stage B disease, Child–Pugh liver function, previous locoregional therapies, underlying liver disease etiology, and duration of follow-up are likely to have influenced survival outcomes across studies. Moreover, differences in national reimbursement policies and early access programs may have affected patient selection and treatment timing. Therefore, the observed similarities and discrepancies among real-world cohorts probably reflect differences in clinical practice patterns and patient characteristics rather than intrinsic differences in the efficacy of the STRIDE regimen itself. Collectively, these findings support the external validity of STRIDE while highlighting the importance of interpreting real-world outcomes within the clinical context of each study population.

The survival and response outcomes observed in our cohort are generally comparable with those reported in previous real-world studies of first-line STRIDE therapy. However, given the retrospective single-arm design and the absence of a control group, these findings should be interpreted as descriptive observations rather than definitive evidence of treatment efficacy.

The incidence of side effects and treatment discontinuation due to side effects was found to be remarkably low, as reported in the literature [30,31]. The favorable safety profile observed in this study should be interpreted with caution. The relatively low incidence of treatment-related adverse events may partly reflect the retrospective study design, the limited sample size, and patient selection through the Turkish national Early Access Program, in which patients were considered eligible for treatment according to predefined clinical criteria. Consequently, the safety outcomes observed in our cohort may not be fully generalizable to the broader population of patients with unresectable hepatocellular carcinoma.

The absence of statistically significant prognostic factors in the present study should not be interpreted as evidence that baseline clinical characteristics lack prognostic relevance. Rather, the relatively small sample size and limited number of outcome events reduced the statistical power of the analyses, increasing the possibility of type II errors. Therefore, the Cox regression analyses should be considered exploratory and hypothesis-generating, and larger prospective studies are warranted to validate potential prognostic factors associated with outcomes following first-line STRIDE therapy.

Although encouraging response rates were observed, the relatively short median overall survival and limited follow-up duration preclude firm conclusions regarding the long-term durability of treatment benefit.

Due to fewer side effects of the STRIDE protocol, new treatment strategies are required to provide longer mPFS and mOS and a high rate of ORR. Emerging immune checkpoint targets, including LAG-3 (lymphocyte-activation gene 3) and TIGIT (T Cell Immunoreceptor with Ig and ITIM Domains), are currently being investigated as potential therapeutic strategies for hepatocellular carcinoma. Although these approaches may further expand the immunotherapy landscape in the future, their role remains investigational. Therefore, larger prospective studies are needed to determine how these novel agents may complement or improve current ICI-based treatment strategies [32,33,34,35].

We reported the first results of tremelimumab/durvalumab treatment in Turkey as a strong side effect. This study has several important limitations. First, its retrospective design introduces the possibility of information bias and unmeasured confounding. Second, the relatively small sample size limited the statistical power of the analyses, reducing the ability to detect potentially meaningful prognostic associations and resulting in imprecise survival estimates. Third, the absence of a control group precludes direct comparisons with other first-line treatment strategies and limits causal inference regarding treatment effectiveness. In addition, the relatively short follow-up period resulted in an immature overall survival estimate, and longer follow-up is required to better characterize long-term outcomes. Selection bias may also have been introduced because patients were treated through the Turkish national Early Access Program and because patients with less than one month of follow-up were excluded from the analysis. Furthermore, response assessments were unavailable for a small proportion of patients because of early treatment discontinuation or insufficient radiological follow-up. Finally, as this study included a selected cohort of patients treated at tertiary oncology centers in Turkey, the findings may not be fully generalizable to broader populations or different healthcare settings. Therefore, our findings should be considered exploratory and hypothesis-generating until they are confirmed in larger, prospective, multicenter studies.

## 5. Conclusions

This multicenter real-world study describes the clinical outcomes observed in Turkish patients with unresectable hepatocellular carcinoma treated with first-line durvalumab plus tremelimumab. The STRIDE regimen appeared to be a feasible treatment option and was generally well tolerated in this selected real-world cohort. However, because of the retrospective design, relatively small sample size, absence of a control group, and limited follow-up, the present study cannot establish treatment efficacy or causal clinical benefit. Therefore, these findings should be considered exploratory and hypothesis-generating and require confirmation in larger, prospective, comparative studies.

## Figures and Tables

**Table 1:** Clinical and demographic features of patients (*n* = 32).

Parameter	
Median Age (Range)—yr	64 (23–85)
Male Sex—no (%)	22 (%68.8)
ECOG Performance Status Score	
0	13 (%40.6)
1	15 (%46.9)
2	4 (%12.5)
Child–Pugh Class/Score	
A/5	20 (%62.5)
A/6	10 (%31.3)
B/7	1 (%3.1)
Other	1 (%3.1)
BCLC Stage	
B	11 (%34.4)
C	21 (%65.6)
Etiology	
HBV	13 (%40.6)
HCV	2 (%6.3)
Non-viral	17 (%53.1)
Macrovascular Invasion	8 (%25)
Extrahepatic Spread	16 (%50)
Lung Metastasis	7 (%21.9)
Bone Metastasis	6 (%18.8)
Lesion Size > 5 cm	24 (%75)
AFP ≥ 400 ng/mL	11 (%34.4)
Cirrhosis	23 (%71.9)
Locoregional Therapies	15 (%46.9)

**Table 2:** Safety profile of patients.

	no, (%)
Any Adverse Events	11 (%34.4)
Grade ≥ 3 Adverse Events	3 (%9.4)
Discontinuation of Treatment	2 (%6.3)

**Table 3:** Adverse events no = 11.

	no, (%)
Pruritus/rash	7 (%21.9)
AST and ALT increased	1 (%3.1)
Adrenal insufficiency	1 (%3.1)
Hypothyroidism	3 (%9.4)
Diarrhea/colitis	3 (%9.4)
Pneumonitis	1 (%3.1)

**Table 4:** Univariable analysis of PFS and OS.

	PFS			OS		
	Hazard Ratio	(95% CI)	*p*-Value	Hazard Ratio	(95% CI)	*p*-Value
Male	0.662	0.275–1.597	0.359	0.664	0.261–1.690	0.39
BCLC stage (B versus C)	1.328	0.539–3.273	0.537	1.198	0.454–3.163	0.716
Etiology (non-viral vs. viral hepatitis)	0.804	0.346–1.868	0.612	0.677	0.274–1.67	0.397
Macrovascular invasion	1.141	0.419–3.107	0.796	1.262	0.454–3.507	0.656
Extrahepatic spread	1.525	0.656–3.546	0.327	1.333	0.539–3.299	0.534
Lung metastasis	0.912	0.336–2.475	0.856	0.596	0.173–2.048	0.411
Bone metastasis	2.552	0.933–6.985	0.068	1.877	0.663–5.313	0.235
Lesion size > 5 cm	1.798	0.66–4.894	0.251	1.596	0.529–4.812	0.407
AFP ≥ 400 ng/mL	1.004	0.382–2.638	0.993	0.634	0.198–2.027	0.442
Cirrhosis	1.827	0.671–4.973	0.238	2.441	0.710–8.389	0.157
Locoregional therapies	0.888	0.383–2.058	0.782	0.606	0.243–1.511	0.282
Adverse events	1.044	0.438–2.491	0.922	0.485	0.172–1.363	0.17

**Table 5:** Best responses—no (%).

Objective	11 (%34.3)
Complete	1 (%3.1)
Partial	10 (%31.3)
Progressive Disease	10 (%31.3)
Stable Disease—no (%)	5 (%15.6)
Disease Control Rate—%	16 (%50)
Exitus	19 (%59.4)

## Data Availability

The data supporting the findings of this study are available from the corresponding author upon request.
